# The Prevalence of Dietary Supplement Usage in Military Aviators

**DOI:** 10.3390/ijerph19095017

**Published:** 2022-04-20

**Authors:** Stefan Sammito, Oliver Maria Erley, Dirk-Matthias Rose, Norbert Güttler

**Affiliations:** 1 German Air Force Centre of Aerospace Medicine, Section Experimental Aerospace Medicine Research, Flughafenstrasse 1, 51147 Cologne, Germany; olivererley@bundeswehr.org (O.M.E.); norbertguettler@bundeswehr.org (N.G.); 2 Occupational Medicine, Faculty of Medicine, Otto von Guericke University of Magdeburg, Leipziger Straße 44, 39120 Magdeburg, Germany; 3 Institute of Occupational, Community and Environmental Medicine, University Medicine, Johannes Gutenberg University of Mainz, Kupferbergterrasse 17, 55116 Mainz, Germany; dirk-matthias.rose@unimedizin-mainz.de

**Keywords:** pilot, human performance, vitamins, stress, nutrition, dietary supplement

## Abstract

Background: The prevalence of dietary supplement (DS) and energy drink (ED) usage in military personnel differs from branch to branch and is between 55% and 76% (higher values in special operations forces). Aviators with highly demanding tasks might be especially interested in using dietary supplements. To date, there are only limited data available for this special profession inside the military. Methods: An internet-based survey was conducted on the prevalence of DS and ED usage, the reasons for their usage and the place of purchase for all wings of the German Armed Forces. Results: Of the 181 pilots who participated in the survey, 34% used DSs and 16% EDs. Usage was linked to sports activities but not to the type of aircraft. DSs were purchased on the internet by 50% of the respondents; mostly protein supplements, magnesium and omega-3fatty acids. Only 42% said they would feel an effect from taking DSs. Conclusions: Although the present study showed that the prevalence of usage was comparable to that of the civilian population, the sources of supply and the range of the substances taken give cause for concern. This calls for education and information campaigns to make the pilots aware of the possible risks to their health.

## 1. Introduction

The term “dietary supplements” (DSs) comprises products that can be purchased without prescription and are used in many countries by a mostly healthy population to supplement their daily diet with vitamins, minerals and trace elements. In the last four decades, the intake of DSs by the US population has increased continuously [1,2]. Currently, about every second adult consumes DSs daily (49–54%) and two thirds of adults consume DSs regularly (64–69%) [1,2]. The annual turnover of DSs regularly exceeds 1 billion USD [3,4]. The intake of DSs differs between the United States and Europe, e.g., in the German population, only 18–50% of all adults take DSs [5,6,7].

Apart from supplementing a diet allegedly lacking in vitamins, minerals and trace elements, amateur and top athletes, in particular, take DSs not only to promote their health, but also to enhance their (physical) performance or to achieve better results in training and regeneration [8,9]. In this group, regular intake of DSs is higher than in the part of the population which is not physically active [1,9] and correlates with frequency of the physical activity [10]. This particularly applies to members of the armed forces. The prevalence of the use of DSs and energy drinks (EDs) differs between each branch of the military service and is between 55% and 76% in soldiers [11]. Soldiers using DSs stated that they spend between 11–50 USD per month on DSs [4,12].

The intake of DSs is viewed critically [4]. When used with the aim of preventing cardiovascular diseases, antioxidant vitamins showed no [13], or even negative, effects [14]. A review conducted to assess the effectiveness of calcium supplementation for improving bone mineral density in healthy children also did not show any significant benefit [15]. Moreover, a majority of the DS examined was contaminated with androgenic and anabolic substances or prohormones [16,17,18].

Among members of the armed forces, the prevalence of DS use is even higher in members of special operations forces [11]. Although data document the intake of DSs in members of the air force, no systematic review exists concerning pilots in particular. Due to the extraordinary professional demands on pilots in military flight operations (e.g., working under high g-load and low ambient pressure), the intake of DSs in this area is of high interest. However, data available for this specific occupational group within the military is so far very limited [19,20,21].

The aim of this survey in the German Armed Forces is (i) to evaluate the intake prevalence of DSs and EDs by military pilots and (ii) to determine the reasons for their use and (iii) the places of their purchase.

## 2. Materials and Methods

An online survey was created using LimeSurvey (LimeSurvey GmbH, Hamburg, Germany) [22]. At the beginning of the survey, all wing commanders were contacted and provided with information about the background of the study and the participation modalities. The commanders were asked to forward the information to all active pilots in their wings with a request to participate. In addition to jet pilots, fixed-wing, rotary-wing, and drone pilots were asked to participate.

The questionnaire (minimum ten items) included personal demographic data concerning gender, age, body size and weight, as well as questions on frequency and type of sport activities, current aircraft type flown and usage of dietary supplements. If such supplements were used, additional questions were asked about the name of the products, the frequency of use and the expected effect, the place where the products had been purchased and the persons who had recommended their use (3 items per DS and 2 additional general items). Further questions were asked on the usage of energy drinks and on how often and why they were consumed (3 items).

All questions could be answered independently, and it was possible to leave any of the questions unanswered. Participation was possible online from 16 November 2020 to 24 January 2021 and by means of any end-user device (workplace computer, private computer, tablet, smartphone); the display of the questionnaire was optimized for all of these devices. On average, it took the participants 3:36 min to complete the survey (min: 0:24 min, max: 28:36 min).

At the beginning of the online survey, all participants were informed about the objective and purpose of the study and about the protection of data privacy. Participation in the study was only possible by means of active continuation (informal consensus). The study had been deliberated and approved by the representative bodies of the Federal Ministry of Defense (reference number: 988 (192-24)). In accordance with the regulations established by the Medical Association of Bavaria, there was no need to obtain an ethic vote for this anonymous survey.

**Figure 1:**
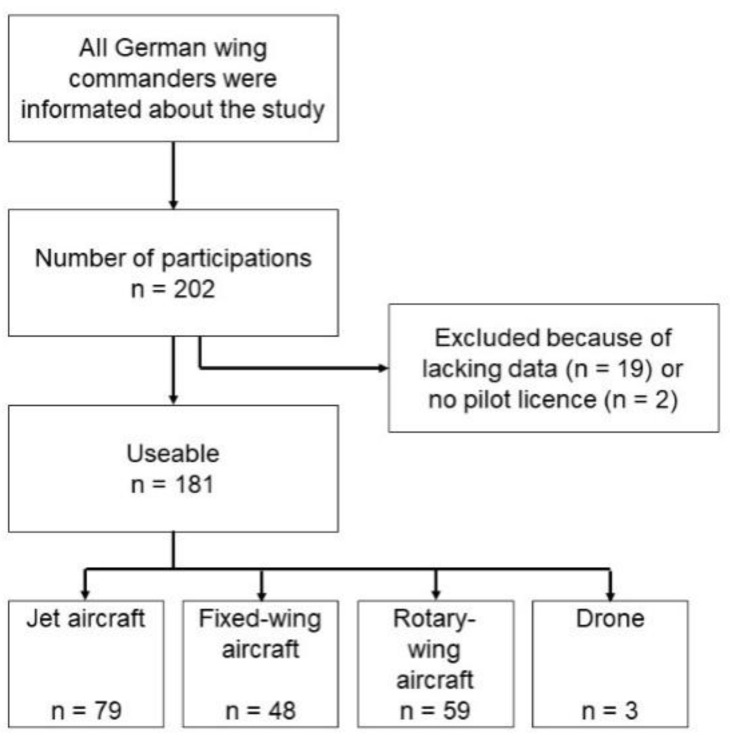
Process of test subject acquisition.

A total of 202 participants were recorded during the survey period. 19 participants were excluded from further analysis due to lacking data, and two had no pilot’s license. In total, 181 pilots (179 male, 2 female; median age: 37 years [min: 24 years, max: 59 years]) were included (see Figure 1).

All data were exported from the LimeSurvey online survey software to IBM SPSS Statistics Version 24 (IBM, Armonk, NY, USA) and analyzed descriptively. Differences between independent groups were examined using a Chi^2^ test or by Kruskal-Wallis-Test, with a significance level of *p* < 0.05.

## 3. Results

Seventy-one of 181 pilots flew jet aircraft, 59 rotary-wing aircraft and 48 fixed-wing aircraft. Three participants were drone pilots, who have not been considered for further analysis due to their small number. According to their own statements, 34% (60/178) of the pilots regularly took DSs and 16% (29/178) regularly consumed EDs. The median number of different DSs taken was two (min: 1, max: 7) and DSs were taken, in median, every day (min: every day, max: once every three months). EDs were consumed at least weekly, but more often by 16% of the pilots; 8% of the pilots used EDs five times or more per week. 64% of the pilots (17/26) did not give a reason for having EDs; 19% (5/26) said they drank EDs to fight fatigue and 15% (4/26) did so because of the taste. 

There was no significant difference between the three types of aircraft with regard to the gender of the pilots, the pilots’ age and self-assessment of their sporting activities, or the prevalence of DS and ED usage (*p* > 0.05 in each case) (see Table 1). There was a significant difference in the intake of DSs between the group with and without sports activities (37% vs. 15%, *p* = 0.032, OR 2.395 [95% CI: 0.949–6.040]). The intake of EDs was comparable in these two groups (17% vs. 12%, *p* = 0.478, OR 1.482 [95% CI: 0.484–4.545]). 

50% of the pilots obtained their DS online, followed by supermarket purchases (27%). Only 17% got their DS from a pharmacy and 10% from their military physician. Most of the pilots decided to take a DS due to personal studies on the subject, while 23% received a recommendation by their military physician and 16% by fellow sportsmen (Table 2 and Table 3). 

Protein supplements (33%), magnesium (22%) and omega-3 fatty acids (20%) were the most frequently taken DSs. However, the range of DSs taken is widely spread (see Table 4).

Forty-two percent of the pilots regularly taking DSs stated that they realized the desired effect; 11 pilots noticed no effect from DSs (18%). In 36% (29/143), DSs were taken to enhance performance, in 22% (31/143) for health reasons and in 20% (29/143) for nutritional reasons. In the remaining cases, the pilots stated other reasons (2%, 3/143) or did not give a reason for taking DSs (20%, 29/143).

## 4. Discussion

Studies about the usage of DSs and of EDs in the highly specialized profession of military aviators are rare and were conducted only in small groups [19,20,21]. With the present study, further insights could be gained, especially with regards to the DSs taken, the reasons for taking them and the sources of supply. This knowledge is of great value if, in the context of preventive education, the advantages and risks of DSs and EDs are explained by professionals.

In this survey, an average of 34% of the respondents (31–42%, depending on the type of aircraft) reported using DSs and 16% EDs regularly. This intake prevalence is well below the intake prevalence of 74% for DSs and 51–79% for EDs referred to in two previously published studies concerning military pilots [19,20,21] and also below the intake prevalence of DSs (74% vs. 55–60%, depending on the branch) [11] or EDs (27%) among soldiers in general [23]. The intake in the analyzed group was not higher than in the general civilian population, with an average DS intake of 18–50% in the adult population [5,6,7], and lower than the intake in the USA (49–54% daily and 64–69% regular consumption [1,2]). Regular surveys on DS intake in the civilian population are rare, and the literature cited is in part from the beginning of this century. This points to a gap in scientific knowledge. It can be assumed that the view on DS intake is changing in the civilian population. 

Although the survey was conducted anonymously, it might be possible that the examined group of active pilots was afraid of personal disadvantages and that pilots who regularly consume DSs therefore did not participate and could therefore be underrepresented. This, as well as the determined intake prevalence, the types of DS and ED consumed, the declared reasons for intake and the sources of supply, should be reason enough to inform pilots about possible advantages and disadvantages of DSs and EDs. In Mannhart’s review about the usefulness of DSs in sports, he stated that only a small fraction of the possible available dietary supplements has a direct or indirect positive influence and that a large number of the DSs can have harmful effects [24,25]. Both the Scientific Committee on Food of the European Commission and the International Olympic Committee concluded that “scientific evidence is lacking or inconsistent in supporting recommendations for nutritional intakes (for sportsmen) beyond the accepted dietary guidelines” [26] and that “the focus should be on consuming a nutrient-rich, well-chosen diet to allow for growth while maintaining a healthy body composition” [27].

There might be a medical indication for an intake (iron supplements (8%) or vitamin D supplements (17%), which would possibly explain why they are widely recommended by physicians (23%). A high number of pilots reported the intake of DSs with ingredients not lacking in the general population and without professional recommendation. The intake of DSs without any need to do so, coupled with possible adverse side-effects, is questionable. The high number of pilots who consumed DSs in association with sport activities (e.g., protein supplements, magnesium, creatine) is concordant with findings in the systematic review by Knapik et al. for military personnel [11]. According to 36% of all pilots, the intake of DSs enhanced their performance. This is an indicator for the motivation that lies behind their consumption. As the survey was conducted anonymously, it cannot be determined retrospectively whether there were real deficiencies or whether the supplements were taken for prophylactic reasons. Unfortunately, the present study cannot provide further insights concerning this matter.

The high proportion of pilots who obtain their DSs either from the internet (50%) or from supermarkets (27%) should be a particular cause for concern, since many DSs are regularly contaminated with prohormones or other androgenic substances [16,17,18]. Taking into account that many of the pilots consume DSs as a result of their own research and a small, but alarming, portion of high-performance athletes do not even know whether or not their DSs are on the list of doping substances [27], active military pilots should be informed and advised about ways to purposefully use DSs. 

Besides the strength of the present study there are some limitations to be taken into account. One of the strengths of the survey is that it was anonymous and could be carried out by the respondents from any internet-enabled device, either at the office or at home. This anonymization was meant to ensure that the barrier to participate was low and that the pilots were not concerned about adverse effects; for example, that they might be grounded because of having completed the questionnaire, or that their lack of supplementation could be interpreted as a sign that they are not taking enough care of their health. Another strength is that all Bundeswehr units with active pilots, including drone pilots, were contacted. Thus, the design of the study did not narrow it down to specific aircraft types and branches of service, but addressed active wings in the Air Force (jet, fixed-wing and rotary-wing aircraft), the Army (rotary-wing aircraft) and the Navy (fixed-wing and rotary-wing aircraft).

The fact that this survey was based on voluntary participation means it is not a representative study of all active military pilots. However, previous surveys among military pilots were mostly of smaller numbers. Since pilots who regularly take dietary supplements may not have participated on purpose because of being afraid that this could have negative repercussions with regards to their pilot’s license, the intake prevalence shown by the study could appear to be too low. At the same time, pilots who do not regularly consume DSs may not have considered themselves as being addressed by the study and may therefore not have participated in the survey. However, these factors should not have a major impact on the study, as the intake prevalence among the entire German population is in a comparable order of magnitude [5,6,7]. Additionally, wing commanders may not have forwarded the information to all active pilots in the wing with the request to participate. Therefore, it is possible that some pilots who wanted to participate did not get this information. On the other hand, this approach was the only way to bring the information into the different wings across Germany.

The reason for the large difference between minimum and maximum time to complete the questionnaire can be explained by the fact that only participants who declared themselves as consuming DSs and/or EDs were asked to provide more information about their specific use of these supplements. Participants who did not use DSs and EDs were only asked to answer 10 questions, which could be done in a short time.

The survey method employed has been shown to be appropriate for answering scientific questions. A goal of further surveys should be to increase the number of participants and maybe link the survey to periodic medical examinations to answer open questions about medically prescribed DSs.

The presented study closes some gaps in scientific literature, especially for the occupational group of military pilots. Further research projects should focus on educational and interventional strategies to reduce the DS intake where DSs are not necessary and to inform pilots about the risks of DSs that are bought from dubitable sources.

## 5. Conclusions

This study is the third published study about intake of DSs worldwide and the first one to also investigate frequencies and reasons for intake of DSs among active German military pilots. There are two corollaries. First, the usage of DSs as a reasonable preventive medical measure should be discussed with a flight surgeon in advance. Second, in view of possible health risks of DSs on one hand, and the finding that many pilots take DSs regularly and that they mainly buy them via the internet or in supermarkets on the other hand, it is necessary for there to be better informational status about DSs in this group. 

## Figures and Tables

**Table 1:** Number of participants for the entire group and for the three types of aircraft (a/c) with median (interquartile-range) for age and number of males, sports activities, prevalence of the intake of dietary supplements and energy drinks.

	Total	Jet a/c	Fixed-Wing a/c	Helicopters	*p*
*n*	178	71 (40%)	48 (27%)	59 (33%)	
*n* males	176	70 (99%)	48 (100%)	58 (98%)	0.680
Age [years]		36 (9)	39 (14)	36 (11)	0.195
Active in sports	152 (85%)	62 (87%)	38 (79%)	52 (88%)	0.357
Intake of dietary supplements	60 (34%)	22 (31%)	20 (42%)	18 (31%)	0.393
Intake of energy drinks	29 (16%)	16 (23%)	5 (10%)	8 (14%)	0.168

**Table 2:** Replies to the question of where dietary supplements are purchased/obtained from, multiple replies were possible, *n* = 60.

Dietary Supplements Purchased/Obtained from	Number
Internet	30 (50%)
Supermarket	16 (27%)
Pharmacy	10 (17%)
Physician	6 (10%)
Sports store	2 (3%)
Gym	1 (2%)
Not specified	10 (17%)

**Table 3:** Replies to the question of who recommended the intake of dietary supplements, multiple replies were possible, *n* = 60.

Intake of Dietary Supplements Recommended by	Number
Self-study	35 (58%)
Unit physician	14 (23%)
Fellow sportsmen	10 (17%)
Internet forums	7 (12%)
Comrades	6 (10%)
Medical personnel other than physicians	4 (7%)
Friends and family	2 (3%)
Sports magazines	2 (3%)
Not specified	13 (22%)

**Table 4:** Groups of dietary supplements used, percentages based on the total number of pilots taking dietary supplements on a regular basis (median every day), sorted by number (descending), *n* = 60.

Dietary Supplement	Number
Protein supplements	20 (33%)
Magnesium	13 (22%)
Omega-3 fatty acids	12 (20%)
Vitamin D	10 (17%)
Creatine	7 (12%)
Multivitamin supplements	7 (12%)
Vitamin B	6 (10%)
Zinc	6 (10%)
Arginine	5 (8%)
Caffeine	5 (8%)
Iron	5 (8%)
Branched-chain amino acids/BCAA	4 (7%)
Calcium	4 (7%)
Selenium	4 (7%)
Coenzyme Q10	1 (2%)
Glutamine	1 (2%)
L-Carnitine	1 (2%)
Potassium	1 (2%)
Taurine	1 (2%)
Vitamin A	1 (2%)
Vitamin E	1 (2%)
Other supplements	4 (7%)
Not specified	8 (13%)

## Data Availability

The data that support the findings of this study are available from the Federal Ministry of Defense. Data are available on reasonable request.

## References

[1] Dickinson A., MacKay D. (2014). Health habits and other characteristics of dietary supplement users: A review. Nutr. J..

[2] Dickinson A., Blatman J., El-Dash N., Franco J.C. (2014). Consumer usage and reasons for using dietary supplements: Report of a series of surveys. J. Am. Coll. Nutr..

[3] Balluz L.S., Kieszak S.M., Philen R.M., Mulinare J. (2000). Vitamin and mineral supplement use in the United States. Results from the third National Health and Nutrition Examination Survey. Arch. Fam. Med..

[4] Greenwood M.R., Oria M. (2008). Use of Dietary Supplements by Military Personnel.

[5] Bodenbach S., Weinkauf B. (1997). Die Einnahme von Vitaminpräparaten in Deutschland. Z. Ernahr..

[6] Reinert A., Rohrmann S., Becker N., Linseisen J. (2007). Lifestyle and diet in people using dietary supplements: A German cohort study. Eur. J. Nutr..

[7] Schwab S., Heier M., Schneider A., Fischer B., Huth C., Peters A., Thorand B. (2014). The use of dietary supplements among older persons in southern Germany—Results from the KORA-age study. J. Nutr. Health Aging.

[8] Braun H., Koehler K., Geyer H., Kleiner J., Mester J., Schanzer W. (2009). Dietary supplement use among elite young German athletes. Int. J. Sport Nutr. Exerc. Metab..

[9] Knapik J.J., Steelman R.A., Hoedebecke S.S., Austin K.G., Farina E.K., Lieberman H.R. (2016). Prevalence of Dietary Supplement Use by Athletes: Systematic Review and Meta-Analysis. Sports Med..

[10] Striegel H., Simon P., Wurster C., Niess A.M., Ulrich R. (2006). The use of nutritional supplements among master athletes. Int. J. Sports Med..

[11] Knapik J.J., Steelman R.A., Hoedebecke S.S., Farina E.K., Austin K.G., Lieberman H.R. (2014). A systematic review and meta-analysis on the prevalence of dietary supplement use by military personnel. BMC Complement. Altern. Med..

[12] Lieberman H.R., Stavinoha T.B., McGraw S.M., White A., Hadden L.S., Marriott B.P. (2010). Use of dietary supplements among active-duty US Army soldiers. Am. J. Clin. Nutr..

[13] Myung S.-K., Ju W., Cho B., Oh S.-W., Park S.M., Koo B.-K., Park B.-J. (2013). Efficacy of vitamin and antioxidant supplements in prevention of cardiovascular disease: Systematic review and meta-analysis of randomised controlled trials. BMJ.

[14] Vivekananthan D.P., Penn M.S., Sapp S.K., Hsu A., Topol E.J. (2003). Use of antioxidant vitamins for the prevention of cardiovascular disease: Meta-analysis of randomised trials. Lancet.

[15] Winzenberg T., Shaw K., Fryer J., Jones G. (2006). Effects of calcium supplementation on bone density in healthy children: Meta-analysis of randomised controlled trials. BMJ.

[16] Geyer H., Parr M.K., Mareck U., Reinhart U., Schrader Y., Schänzer W. (2004). Analysis of non-hormonal nutritional supplements for anabolic-androgenic steroids—Results of an international study. Int. J. Sports Med..

[17] Maughan R.J. (2005). Contamination of dietary supplements and positive drug tests in sport. J. Sports Sci..

[18] Geyer H., Parr M.K., Koehler K., Mareck U., Schänzer W., Thevis M. (2008). Nutritional supplements cross-contaminated and faked with doping substances. J. Mass Spectrom..

[19] Bukhari A.S., Caldwell J.A., DiChiara A.J., Merrill E.P., Wright A.O., Cole R.E., Hatch-McChesney A., McGraw S.M., Lieberman H.R. (2020). Caffeine, Energy Beverage Consumption, Fitness, and Sleep in U.S. Army Aviation Personnel. Aerosp. Med. Hum. Perform..

[20] Sather T.E., Woolsey C.L., Delorey D.R., Williams R.D. (2018). Energy Drink and Nutritional Supplement Beliefs Among Naval Aviation Candidates. Aerosp. Med. Hum. Perform..

[21] Bukhari A.S., Caldwell J.A., DiChiara A.J., Merrill E.P., Wright A.O., Lieberman H.R. (2017). Army Aircrew Members perspectives on use of dietary supplements and energry drinks. Aerosp. Med. Hum. Perform..

[22] Lime Survey GmbH (2012). Lime Survey: An Open Source Survey Tool.

[23] Knapik J.J., Austin K.G., McGraw S.M., Leahy G.D., Lieberman H.R. (2017). Caffeine consumption among active duty United States Air Force personnel. Food Chem. Toxicol..

[24] Mannhart C. (2003). State of the art of nutritional supplements in sport. Schweiz. Z. Sportmed. Sporttraumatologie.

[25] Maughan R.J., Burke L.M., Dvorak J., Larson-Meyer D.E., Peeling P., Phillips S.M., Rawson E.S., Walsh N.P., Garthe I., Geyer H. (2018). IOC consensus statement: Dietary supplements and the high-performance athlete. Br. J. Sports Med..

[26] European Commission, Scientific Committee on Food (2001). Report of the Scientific Committee on Food on Composition and Specification of Food Intended to Meet the Expenditure of Intense Muscular Effort, Especially for Sportsmen.

[27] Sundgot-Borgen J., Berglund B., Torstveit M.K. (2003). Nutritional supplements in Norwegian elite athletes--impact of international ranking and advisors. Scand. J. Med. Sci. Sports.

